# Cost-effectiveness analysis of toripalimab for metastatic or recurrent triple-negative breast cancer

**DOI:** 10.3389/fonc.2023.1268584

**Published:** 2024-01-18

**Authors:** Jiangbo Shao, Cuiping Zhan, Chunxiang Jin, Ying Jin

**Affiliations:** ^1^ Department of Ultrasound, China–Japan Union Hospital of Jilin University, Changchun, Jilin, China; ^2^ Department of Breast Surgery, General Surgery Center, The First Hospital of Jilin University, Changchun, Jilin, China

**Keywords:** cost-effectiveness analysis, toripalimab, PD-L1 positive, triple negative breast cancer, immunotherapy

## Abstract

**Background:**

Toliparibizumab in combination with nab-paclitaxel (T+N) has excellent efficacy inmetastatic or recurrent triple-negative breast cancer (TNBC), but the optimal choice of sequence of therapy is unclear given the trade-offs between quality of life and cost. Cost-effectiveness analyses can quantify these tradeoffs, leading to more informed decisions. Our objective was to assess the societal cost-effectiveness of the T+N regimen for metastatic or recurrent TNBC.

**Methods:**

Clinical data were extracted from a multicenter, randomized, double-blind trial, TORCHLIGHT (NCT04085276). Patients were randomized into the T+N group or placebo plus nab-paclitaxel (P+N) group. 531 patients from 53 study locations were randomly assigned (T+N, n=353; P+N, n=178) into intend to treat (ITT) population; 200 and 100 patients, respectively had programmed death protein 1 (PD-L1) positive TNBC. A Markov model was established with a 21-day cycle length. Costs were acquired from local hospitals, effect parameters included quality-adjusted life year (QALY) and incremental cost-effectiveness ratio (ICER).

**Results:**

The cost differences were 47,538.3 CNY in ITT population (T+N, 143,725.67 CNY; P+N group, 96,187.37 CNY) and 29,258.84 CNY in PD-L1+ subgroup (T+N, 100,128.28 CNY; P+N group, 70,869.45 CNY). Meanwhile, the IEs were 0.03409 in the ITT population (T+N, 0.55323 QALY; P+N, 0.51914 QALY) and 0.03409 in the PD-L1+ subgroup (T+N, 0.42327 QALY; P+N, 0.37628 QALY). The ICERs between T+N and P+N groups were 1,394,548.41 CNY/QALY in the ITT population and 622,663.98 CNY/QALY in the PD-L1+ subgroup. We also analyzed the cost-effectiveness of toripalimab could be received in the Chinese medical insurance catalog. If toripalimab could be reimbursed at an 80% rate, the cost differences were changed to 16,598.99 CNY in ITT population (T+N, 112,786.36 CNY; P+N group, 96,187.37 CNY) and 7,704.58 CNY in PD-L1+ subgroup (T+N, 78,574.03 CNY; P+N group, 70,869.45 CNY). Meanwhile, the IEs remained unchanged. The ICERs between T+N and P+N groups were changed to 486,935.82 CNY/QALY in the ITT population and 163,962.96 CNY/QALY in the PD-L1+ subgroup. Sensitivity analyses indicated the stability of the model and the impact of utility.

**Conclusion:**

At current drug prices, the T+N group is not more cost-effective than the P+N group, but after incorporating toripalimab into medical insurance, the T+N group will be more cost-effective for patients with PD-L1+ metastatic or recurrent triple-negative breast cancer.

## Introduction

Breast cancer (BC) has surpassed lung cancer as the most often diagnosed cancer and the world’s fifth leading cause of cancer mortality. In 2070, there are projected to be 4.4 million cases (1, 2). Breast cancer poses a severe danger to women’s lives as it accounts for approximately 24.5% of all cancer cases and 15.5% of cancer deaths among women (3). Most nations in the globe will have the highest incidence and fatality rates by 2020 (1).

TNBC has a poor prognosis and lacks targets that can be used for Targeted therapy. Therefore, chemotherapy has been the main treatment method for many years and new treatment breakthroughs are urgently needed (4, 5). Immune checkpoint inhibitors (ICI) treatment is a milestone in the treatment of malignant tumors. Its clinical application has completely changed the treatment methods of several cancers, including lung cancer and melanoma, and the prognosis and quality of life of patients have been significantly improved (6). With the in-depth study of the TNBC immune microenvironment, it is found that compared with other breast cancer subtypes, Tumor-infiltrating lymphocytes, PD-L1 expression, and tumor mutation load levels are higher (7–9). These characteristics suggest that immunotherapy may be a new therapeutic hope for TNBC (4).

In a recent meta-analysis (10) pooling six published clinical studies of immunotherapy for triple-negative breast cancer, immunotherapy based on programmed death protein 1 (PD-1)/PD-L1 inhibitors safely improved progression-free survival in patients with unresectable locally advanced or metastatic TNBC (p<0.001), but did not affect overall survival (OS) (p = 0.144). However, there are many complex molecular subtypes of TNBC, and there are still many unresolved questions that pose a great challenge for treatment (11, 12), and can we screen for a more beneficial population by changing the combination regimen, altering the timing of the application of immunotherapy, and applying appropriate biomarkers (9, 13).

Toripalimab (Tuoyi™) is a selective, recombinant, humanized monoclonal antibody against PD-1. Toripalimab has shown excellent anti-tumor effects in a wide range of tumors including melanoma, lung cancer, and uroepithelial cancer, and has been well tolerated by patients.

Recently, Jiang et al. conducted the publication of the results of the TORCHLIGHT study (NCT04085276) (14). This study was the first to use toripalimab in breast cancer patients and the investigators gave an oral presentation at ASCO 2023 (14). This was a randomized, double-blind, placebo-controlled, multi-center, phase III clinical study designed to compare the efficacy and safety of toripalimab in combination with injectable albumin in patients with a first diagnosis of stage IV or relapsed metastatic TNBC paclitaxel versus placebo combination chemotherapy in patients with first diagnosis of stage IV or recurrent metastatic TNBC.

Compared to chemotherapy, treatment with the toripalimab combination significantly prolonged progression-free survival (PFS) in the PD-L1-positive population, while the secondary endpoint of OS showed a trend towards a significant benefit in both the full population and the PD-L1-positive population. The toripalimab safety data were consistent with known risks and no new safety hazards were identified.

Based on the first disclosure of the study results, whether the application of toripalimab can bring economic benefits to TNBC patients. This study was conducted to analyze the relevant data, to give inspiration to clinicians and relevant departments to bring more benefit to patients. Cost-effectiveness analyses have been used to quantify the clinical benefits as well as the potential cost associated with the new therapies. Our study aims to investigate the cost-effectiveness of toripalimab plus nab-paclitaxel treatment for metastatic or recurrent TNBC.

## Methods

**Figure 1:**
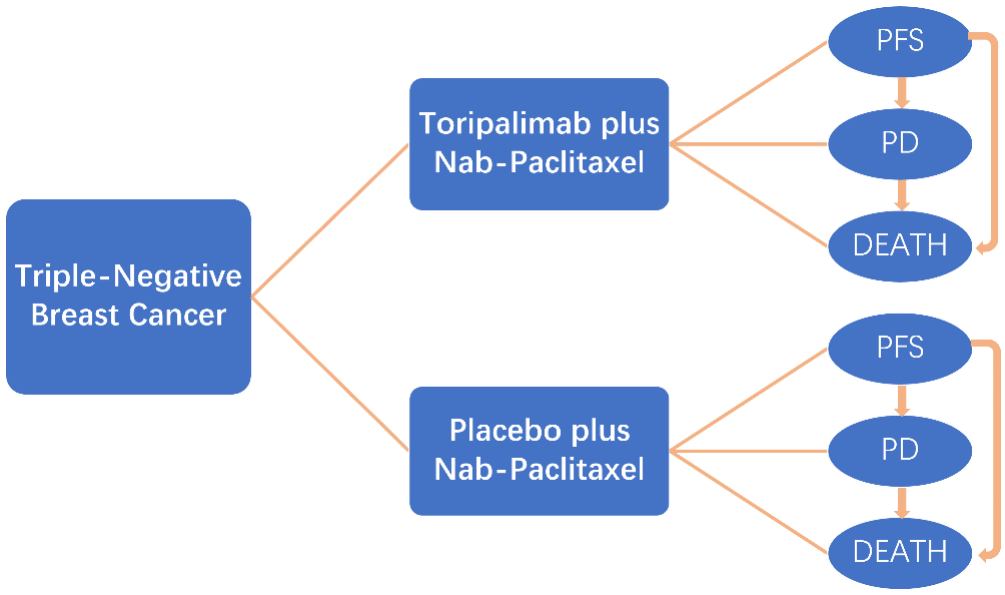
Markov model for triple-negative breast cancer. A Markov model comprising three health states (progression-free state, progressive disease, and death) was built.

This economic analysis was based on the Markov model and did not require approval from an institutional review board (**Figure 1**).

### Clinical parameters

Clinical patient characteristics and outcomes were from the TORCHLIGHT (NCT04085276) (14). TORCHLIGHT was a multicenter, randomized, double-blind study that would evaluate the efficacy and safety of T+N group compared with P+N group for first/second-line treatment of metastatic or recurrent TNBC.

The criteria for enrolling patients include the following:

Inclusion Criteria:

Metastatic or recurrent TNBC;

(1) Histologically confirmed diagnosis of TNBC characterized by estrogen-receptor negative (ER-), progesterone receptor negative (PR-), and human epidermal growth factor-2 receptor negative (HER2-);
(2) Eligible for taxane monotherapy;
(3) No more than one line of chemotherapy in a metastatic setting;
(4) Eastern Cooperative Oncology Group (ECOG) performance status of 0 or 1;
(5) Life expectancy of 12 weeks or more;
(6) At least one measurable lesion per RECIST v1.1;

Exclusion Criteria:

Prior treatment with taxane as first-line treatment;

(1) Prior treatment with PD-1 antibody, PD-L1 antibody, PD-L2 antibody, or CTLA4 antibody (or any other antibody acting on T cell co-stimulation or checkpoint pathway);
(2) MRI assessment during screening or previous imaging studies confirmed active or untreated brain metastases. Patients previously treated with local treatment of brain metastases have been stable for ≥ 1 month, and have stopped systemic hormonal therapy (>10 mg/d prednisone or equivalent) > 4 weeks before randomization can participate in the study;
(3) Meningeal carcinomatosis;
(4) Pregnancy or lactation;
(5) Active hepatitis B or hepatitis C.

531 patients from 53 study locations were randomly assigned (T+N, n=353; P+N, n=178) into ITT population; 200 and 100 patients, respectively had PD-L1 positive TNBC. Patients were randomly assigned 2:1 to the T+N group and P+N group. In the T+N group, toripalimab (240mg, i.v., q3w) plus nab-paclitaxel (125 mg/m^2^, i.v., day1, day8, q3w) was administered every 21 days until disease progression or intolerable side effects. In the P+N group, toripalimab was replaced by a placebo, and other treatments were identical to those of the T+N group. Coprimary endpoints were centrally confirmed PFS and OS.

Among the ITT population, PFS was improved with the T+N group (hazard ratio [HR], 0.77; 95% CI, 0.60 to 0.99; stratified log-rank P=0.0445). The median PFS was 8.4 months (7.0 to 9.8 months) in the T+N group and 6.9 months (5.5 to 8.2 months) in the P+N group. The OS HR was 0.69 (95% CI, 0.51 to 0.93; P=0.0145). The median OS was 33.1 months (28.1 months to NE) in the T+N group and 23.5 months (18.6 to 28.9 months) in the P+N group. The OS HR was 0.69 (95% CI, 0.51 to 0.93; P=0.0145). Among the PD-L1+ subgroup, PFS was improved with the T+N group (hazard ratio [HR], 0.65; 95% CI, 0.47 to 0.91; stratified log-rank P=0.0102). The median PFS was 8.4 months (6.9 to 10.9 months) in the T+N group and 5.6 months (5.0 to 7.2 months) in the P+N group. The OS HR was 0.62 (95% CI, 0.41 to 0.91; P=0.0148). The median OS was 32.8 months (26.9 months to NE) in the T+N group and 19.5 months (15.1 to 32.2 months) in the P+N group.

### Markov model

We established a Markov model with Treeage Pro 2011 (Treeage Software Inc., Williamstown, MA) to evaluate the cost-effectiveness of different treatment strategies. The Markov model of each therapeutic involved three mutually transferable health states: PFS, progressive disease (PD), and death. All patients were defined in the PFS state in the beginning and subsequently survived or died; patients who survived either remained in the PFS state or transferred to the PD state. Patients who transferred to the PD state either remained or died. The cycle length of transition probabilities was 21 days based on the period of therapy. The cost and utility values were calculated at a 3% annual discount rate (15). The primary endpoints of the cost-effectiveness analysis were QALY and incremental cost-effectiveness ratio (ICER). Secondary endpoints were the average cost-effectiveness ratio (average CE) and net benefit (willing-to-pay [WTP] benefit-costs).

### Costs and utilities

This study only considers direct medical costs, including drug costs, test and examination costs, doctor’s diagnosis and treatment costs, material costs, bed costs, nursing costs, and treatment costs for common grade 3 or 4 adverse events. Tests cost included computed tomography, magnetic resonance imaging, single-photon emission computed tomography, and other blood tests.

Here, we need to explain that, the experimental data of this clinical trial has not been fully disclosed, and we have not found the original experimental data for common grade 3 or 4 adverse events, so we did not analyze it in this section. Other drug costs and test costs were extracted from the local hospital in China (**Table 1**). All data in USD are converted into RMB at the exchange rate of 7.2199 (July 19, 2023).

**Table 1:** Model economic parameters and the range of the sensitivity analysis.

Variables	Baseline value(range)	Distribution	Source
Cost (CNY)			
Toripalimab(240mg)	2,101(1,470.7-2,731.3)	Gamma	Local charge
Nab-Paclitaxel(100mg)	747(522.9-971.1)	Gamma	Local charge
Gemcitabine(1000mg)	1,838.4(1,286.88-1,672.95)	Gamma	Local charge
Carboplatin(100mg)	792(554.4-1,029.6)	Gamma	Local charge
Routine follow-up cost per cycle	430(301-559)	Gamma	Local charge
Best supportive care per cycle	2,609(1,826.3-3,391.7)	Gamma	Local charge
Laboratory testing	2,289(1,602.3-2,975.7)	Gamma	Local charge
Tumor imaging	1,678(1,174.6-2,181.4)	Gamma	Local charge
Utility value			
PFS	0.72(0.576-864)	Beta	(16)
PD	0.45(0.36-0.54)	Beta	(16)
Body surface area(m2)	1.6(1.28-1.92)	Beta	(17)
Discount rate (%)	3	Beta	(18)

PFS, progression-free survival; PD, progressive disease.

### Sensitivity analysis

We performed a one-way sensitivity analysis to assess the impact of each parameter on model outputs. The ranges were set as ±20% for utilities and ±30% for costs (**Table 1**). For the probabilistic sensitivity analysis, a Monte Carlo simulation was performed with 1000 iterations, and each parameter was fitted to a specific distribution: a beta distribution for utilities and lognormal distribution for costs (16). The WTP threshold was set to three times the per capita GDP of China in 2021: 243,000 CNY/QALY. The results are described as cost-effectiveness acceptability curves.

## Results

Total costs incurred were 143,725.67 CNY in the T+N group and 96,187.37 CNY in the P+N group, resulting in a cost difference of 47,538.3 CNY in the ITT population. Meanwhile, the QALY in T+N was 0.03409 higher than that in the P+N group (T+N, 0.55323 QALY; P+N, 0.51914 QALY). The ICER between T+N and P+N groups was 1,394,548.41 CNY/QALY in the ITT population (**Table 2**). The results show that the T+N group was not more cost-effective than the P+N group.

**Table 2:** A cost-effectiveness analysis.

Strategy	QALY	Cost (CNY)	IE	IC	ICER(CNY/QALY)
ITT population					
T+N	0.55323	143,725.67	0.03409	47,538.30	1,394,548.41
P+N	0.51914	96,187.37	0	0	0
PD-L1+ subgroup					
T+N	0.42327	100,128.28	0.04699	29,258.84	622,663.98
P+N	0.37628	70,869.45	0	0	0

QALY, quality-adjusted life year; IE, incremental effect; IC, incremental cost; ICER, incremental cost-effectiveness ratio.

In the PD-L1+ subgroup, total costs incurred were 100,128.28 CNY in the T+N group and 70,869.45 CNY in the P+N group, resulting in a cost difference of 29,258.84 CNY. Meanwhile, the QALY in the T+N group was 0.04699 higher than that in the P+N group (T+N, 0.42327 QALY; P+N, 0.37628 QALY). The ICER between T+N and P+N groups was 622,663.98 CNY/QALY in the PD-L1+ subgroup (**Table 2**). The results show that T+N was also not cost-effective.

In our analysis, it was found that the T+N group was not significantly cost-effective compared to the chemotherapy group (P+N), which may be due to the drug price of toripalimab, which affects the cost-effectiveness of the T+N group. If toripalimab could be included in the Chinese medical insurance catalog, the economic burden of patients will be reduced. It was assumed that 80% of healthcare costs would be paid by healthcare payers (19).

Total costs incurred were 112,786.36 CNY in the T+N group and 96,187.37 CNY in the P+N group, resulting in a cost difference of 16,598.99 CNY in the ITT population. Meanwhile, the QALYs were the same as before. The ICER between T+N and P+N groups was 486,935.82 CNY/QALY in the ITT population (**Table 3**). In the PD-L1+ subgroup, total costs incurred were 78,574.03 CNY in T+N and 70,869.45 CNY in the P+N group, resulting in a cost difference of 7,704.58 CNY. The ICER between T+N and P+N groups was 163,962.96 CNY/QALY in the PD-L1+ subgroup (**Table 3**). The results show that T+N was cost-effective in the PD-L1+ subgroup.

**Table 3:** Cost-effectiveness analysis after toripalimab is included in medical insurance.

Strategy	QALY	Cost (CNY)	IE	IC(CNY)	ICER(CNY/QALY)
ITT population					
T+N	0.55323	112,786.36	0.03409	16,598.99	486,935.82
P+N	0.51914	96,187.37	0	0	0
PD-L1+ subgroup					
T+N	0.42327	78,574.03	0.04699	7,704.58	163,962.96
P+N	0.37628	70,869.45	0	0	0

QALY, quality-adjusted life year; IE, incremental effect; IC, incremental cost; ICER, incremental cost-effectiveness ratio.

**Figure 2:**
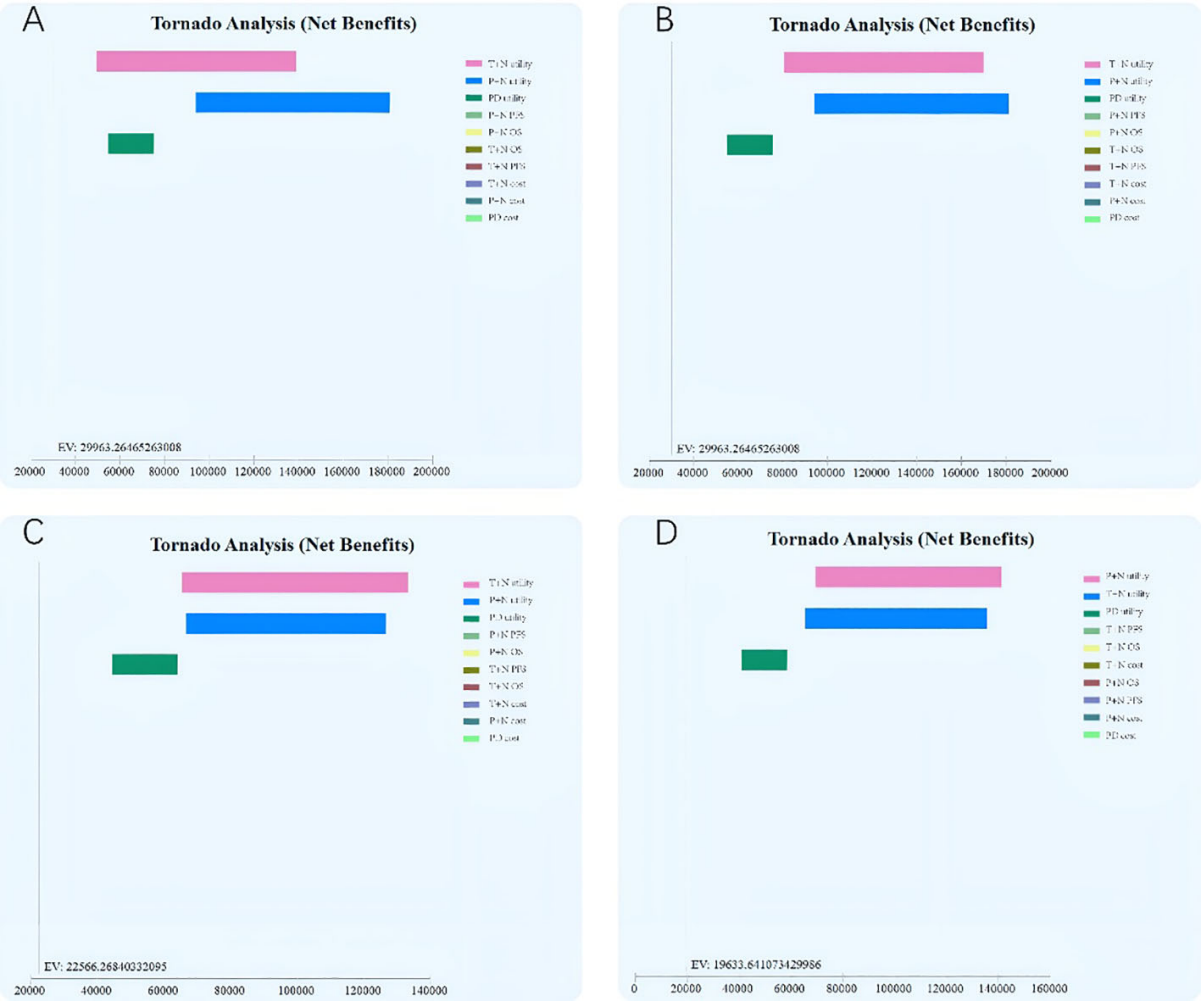
One-way sensitivity analysis tornado diagram of the incremental cost–effectiveness ratio (cost per quality-adjusted life-year gained) of toripalimab plus chemotherapy versus chemotherapy.

Results of the one-way sensitivity analysis indicated that the values of T+N utility, P+N utility, and PD utility were the most influential parameter both in the ITT population and the PD-L1+ subgroup (**Figure 2**). A probabilistic sensitivity analysis with Monte Carlo simulations revealed that T+N is cost-effective compared with P+N in the PD-L1+ subgroup after toripalimab is included in medical insurance.

**Figure 3:**
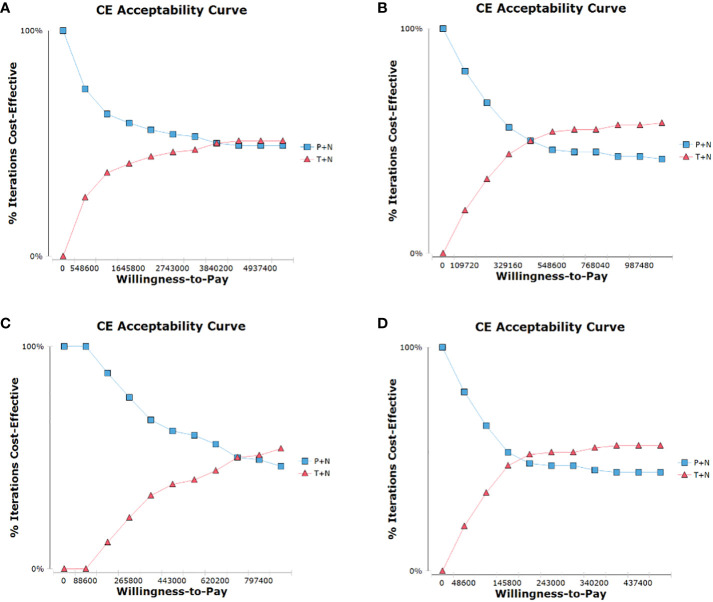
Cost-effectiveness acceptability curve for comparison among various treatment regimens. **(A)**, ITT population, **(B)**, PD-L1+ subgroup, **(C)**, ITT population after toripalimab is included in medical insurance, **(D)**, PD-L1+ subgroup after toripalimab is included in medical insurance; CE, cost-effectiveness.

In the probabilistic sensitivity analysis, the cost-effectiveness acceptability curve (**Figure 3**) showed T+N group achieved economic benefits even if the WTP was set at ¥3,840,200/QALY in the ITT population, but could achieve economic benefits if the WTP was set at ¥438,880/QALY after toripalimab is included in medical insurance. T+N group achieved economic benefits even if the WTP was set at ¥708,800/QALY in the PD-L1+ subgroup, but could achieve cost-effectiveness benefits if the WTP was set at ¥171,600/QALY after toripalimab is included in medical insurance.

## Discussion

Over the past few decades, breast cancer has become the most common type of cancer among women. Despite advances in basic research, the lack of targeted therapy has limited treatment options for breast cancer and is accompanied by a wide range of side effects. Recent advances in targeted therapies have provided a more targeted and efficient treatment option for breast cancer. Some targeted agents, either independently or in combination with other drugs, have received Food and Drug Administration (FDA) approval for the treatment of different breast cancer subtypes, and many of them are in clinical trials. Immunotherapy is expected to be used for the immunotherapy of TNBC patients. Some immune checkpoint blockers used in combination with other drugs have received FDA approval for the treatment of TNBC.

ICI therapy is a milestone in the treatment of malignant tumors, with significant improvements in patient prognosis and quality of life. ICI-based immunotherapy for BC has focused on TNBC, which is the most difficult type of BC to treat. Meanwhile, TNBC patients with higher levels of PD-L1 expression, tumor mutation burden (TMB), and tumor-infiltrating lymphocytes may be more likely to benefit from ICI therapy compared to patients with other BC subtypes (5, 20). Anti-pd -1 monoclonal antibodies have been shown to have anti-tumor activity in the first-line treatment of PD-L1-positive metastatic TNBC and the neoadjuvant treatment of patients with high-risk early TNBC (21, 22). The therapeutic value of immunotherapy has also been investigated in second-line and There are no satisfactory predictors of efficacy. Detection of PD-L1 expression on the surface of tumor cells by immunohistochemical methods is promising.

Toripalimab is the first domestically produced anti-tumor PD-1 antibody. Toripalimab, which has potent anti-tumor effects, was first approved for the second-line treatment of metastatic melanoma in China in December 2018, and for the treatment of patients with recurrent/metastatic nasopharyngeal carcinoma (RM-NPC) who have previously failed second-line or more systemic therapy in China in February 2021, toripalimab is a humanized IgG4K monoclonal antibody against PD-1, which has provided significant clinical efficacy and a favorable safety profile in various solid tumours.

In our study, we established a Markov model to assess the cost-effectiveness of toripalimab plus nab-paclitaxel compared with placebo plus nab-paclitaxel for first/second-line treatment of metastatic or recurrent TNBC. We discovered that, while the T+N group improved patients’ PFS and OS, it was not cost-effective for either the ITT population or the PD-L1+ subgroup. This might be owing to the high cost of toripalimab. When we added toripalimab in the health insurance catalog and reimbursed it at 80%, we discovered that the T+N group was still not cost-effective in the ITT population, but it was significantly cost-effective in the PD-L1+ subgroup. As a result, we recommend that the indication for toripalimab be expanded to include PD-L1+ TNBC in the medical insurance catalog. This will not only enhance patients’ OS and PFS, but will also not place a significant financial burden on them.Utility costs were the characteristics that had the biggest impact in the one-way sensitivity analysis. Based on the practical rates found in the clinical studies, utilities were modified. Despite the fact that these utilities were taken from published literature, a current multinational research that offered utilities for various demographics was chosen.

In conclusion, this study has some limitations. First, we exclusively focus on moderate to severe adverse events recorded in clinical trials; moreover, the utility employed in our analysis may not accurately reflect the total health effect experienced by patients getting these medicines or their choice for certain parts of therapy. Our model does not take into account minor adverse events that have significance for patients, patients who experience distinct multiple adverse events from different treatments, or patients who have strong treatment preferences unrelated to adverse events. This is primarily due to the paucity of data that supports this assumption. Second, the pricing of pharmaceuticals is a dynamic process that is influenced by a number of factors, including the emergence of new rivals and patent protection. We have a very sensitive model to changes in costs.

## Data availability statement

The original contributions presented in the study are included in the article/supplementary material. Further inquiries can be directed to the corresponding authors.

## Author contributions

JS: Writing – original draft. CZ: Writing – review & editing. CJ: Writing – original draft. YJ: Writing – review & editing.
